# An on-ice aerial survey of the Kane Basin polar bear (*Ursus maritimus*) subpopulation

**DOI:** 10.1007/s00300-021-02974-6

**Published:** 2021-11-22

**Authors:** Øystein Wiig, Stephen N. Atkinson, Erik W. Born, Seth Stapleton, Todd Arnold, Markus Dyck, Kristin L. Laidre, Nicholas J. Lunn, Eric V. Regehr

**Affiliations:** 1 Natural History Museum, University of Oslo, Blindern, P.O. Box 1172, N-0318 Oslo, Norway; 2 Wildlife Research Section, Department of Environment, Government of Nunavut, P.O. Box 209, Igloolik, NU X0A 0L0 Canada; 3 Greenland Institute of Natural Resources, P.O. Box 570, 3900 Nuuk, Greenland; 4 Field Conservation Program, Minnesota Zoo, Apple Valley, MN 55124 USA; 5 Department of Fisheries, Wildlife, and Conservation Biology, University of Minnesota, St. Paul, MN 55108 USA; 6 Polar Science Center, Applied Physics Laboratory, University of Washington, Seattle, WA USA; 7 Environment and Climate Change Canada, CW-422 Biological Sciences Building, University of Alberta, Edmonton, AB T6G 2E9 Canada

**Keywords:** Abundance, Aerial survey, Distance sampling, Kane Basin, North Water Polynya, Polar bear, *Ursus maritimus*

## Abstract

There is an imminent need to collect information on distribution and abundance of polar bears (*Ursus maritimus*) to understand how they are affected by the ongoing decrease in Arctic sea ice. The Kane Basin (KB) subpopulation is a group of high-latitude polar bears that ranges between High Arctic Canada and NW Greenland around and north of the North Water polynya (NOW). We conducted a line transect distance sampling aerial survey of KB polar bears during 28 April–12 May 2014. A total of 4160 linear kilometers were flown in a helicopter over fast ice in the fjords and over offshore pack ice between 76° 50′ and 80° N′. Using a mark-recapture distance sampling protocol, the estimated abundance was 190 bears (95% lognormal CI: 87–411; CV 39%). This estimate is likely negatively biased to an unknown degree because the offshore sectors of the NOW with much open water were not surveyed because of logistical and safety reasons. Our study demonstrated that aerial surveys may be a feasible method for obtaining abundance estimates for small subpopulations of polar bears.

## Introduction

A growing body of evidence indicates that polar bears (*Ursus maritimus*) are being affected by long-term climate change, primarily through reductions in the availability and quality of their sea ice habitat (e.g. Regehr et al. 2007; Rode et al. 2010; Atwood et al. 2015; Lunn et al. 2016; Obbard et al. 2016; Laidre et al. 2018a, b; 2020a, b). However, responses to climate change have been predicted (Derocher et al. 2004; Amstrup 2011; Stirling and Derocher 2012; Regehr et al. 2016) and observed (e.g. Rode et al. 2014; Regehr et al. 2018; Laidre et al. 2018a, b, 2020a, b) to differ across the species’ circumpolar range. Subpopulations in southern portions of the range, where the annual sea ice melts completely during summer and autumn (e.g. polar bears in the Seasonal sea ice ecoregion, Amstrup et al. 2008), are predicted to be the first to experience negative effects, such as reduced body condition, reproductive performance, and survival (e.g. Stirling and Derocher 2012). In contrast, subpopulations in northern portions of the range (e.g. the Archipelago ecoregion, Amstrup et al. 2008) may initially show positive effects as these regions shift from multi-year sea ice to thinner, annual sea ice and a longer period of open water (Hamilton et al. 2014). A longer period of open water may lead to increased marine productivity that ultimately also affect higher levels of the food web (Laidre et al. 2020a), including ringed (*Pusa hispida*) and bearded seals (*Erignathus barbatus*) that are the primary prey of polar bears; these habitat changes may enhance per capita food availability for some multi-year sea ice subpopulations of polar bears (Derocher et al. 2004; Stirling and Derocher 2012; Laidre et al. 2020a).

A straight-forward way to better understand the status of polar bears is through quantitative information on the number of animals in the subpopulation and the trend in that number (Vongraven et al. 2012). Given variability in timing and direction of responses exhibited by polar bears to climate change, research and monitoring are needed range-wide to implement state-dependent (i.e., dependent on current conditions) management (Regehr et al. 2017a, b), assess population viability (Lunn et al. 2016), and understand the species’ ability to adapt to changing conditions (Vongraven et al. 2012). Accurate and timely information is essential for adaptive management measures such as harvest level adjustments or supplemental feeding, and for detecting sudden changes in subpopulation status (Derocher et al. 2013).

The Kane Basin (KB) and neighboring Norwegian Bay subpopulations in the Canadian High Arctic (i.e. the Archipelago ecoregion) are among the smallest and most northerly distributed of all polar bear subpopulations (PBSG 2018). Habitat available to polar bears in the KB subpopulation is characterized by a mixture of multi-year and annual sea ice that is available to the bears year-round (Hamilton et al. 2014; Stern and Laidre 2016; Laidre et al. 2020b). Based on the observed long-term trend of decreasing multi-year ice and increasing ice-free periods (Stern and Laidre 2016), KB has been shown to be experiencing positive effects from climate change (Derocher et al. 2004; Stirling and Derocher 2012; Laidre et al. 2020b). Recent ecological studies found evidence of range expansions and improved body condition indicating an overall increase in marine productivity to the benefit of seals and polar bears (Laidre et al. 2020b). However, the high cost, logistical challenges, and low density of bears make on-going monitoring of the status of the KB subpopulation difficult. Thus, development of cost-effective methods for monitoring small, remote subpopulations of polar bears has become an area of interest for management agencies across the Arctic (Vongraven et al. 2012; Polar Bear Range States 2015).

Under the auspices of the Canada-Greenland Joint Commission on Polar Bear, a multi-year mark-recapture (MR) study (2012–2014) was conducted to estimate the size of the KB subpopulation (SWG 2016). In 2014, we conducted an aerial survey during the final year of this MR study with the main objective being to evaluate the feasibility of estimating abundance using an aerial survey flown over springtime sea ice in the KB subpopulation. A comprehensive comparison of the estimates of abundance obtained from the MR study and the aerial survey in KB as well as management implications was presented in SWG (2016) and is under preparation for publication.

## Materials and methods

### Study area

**Fig. 1:**
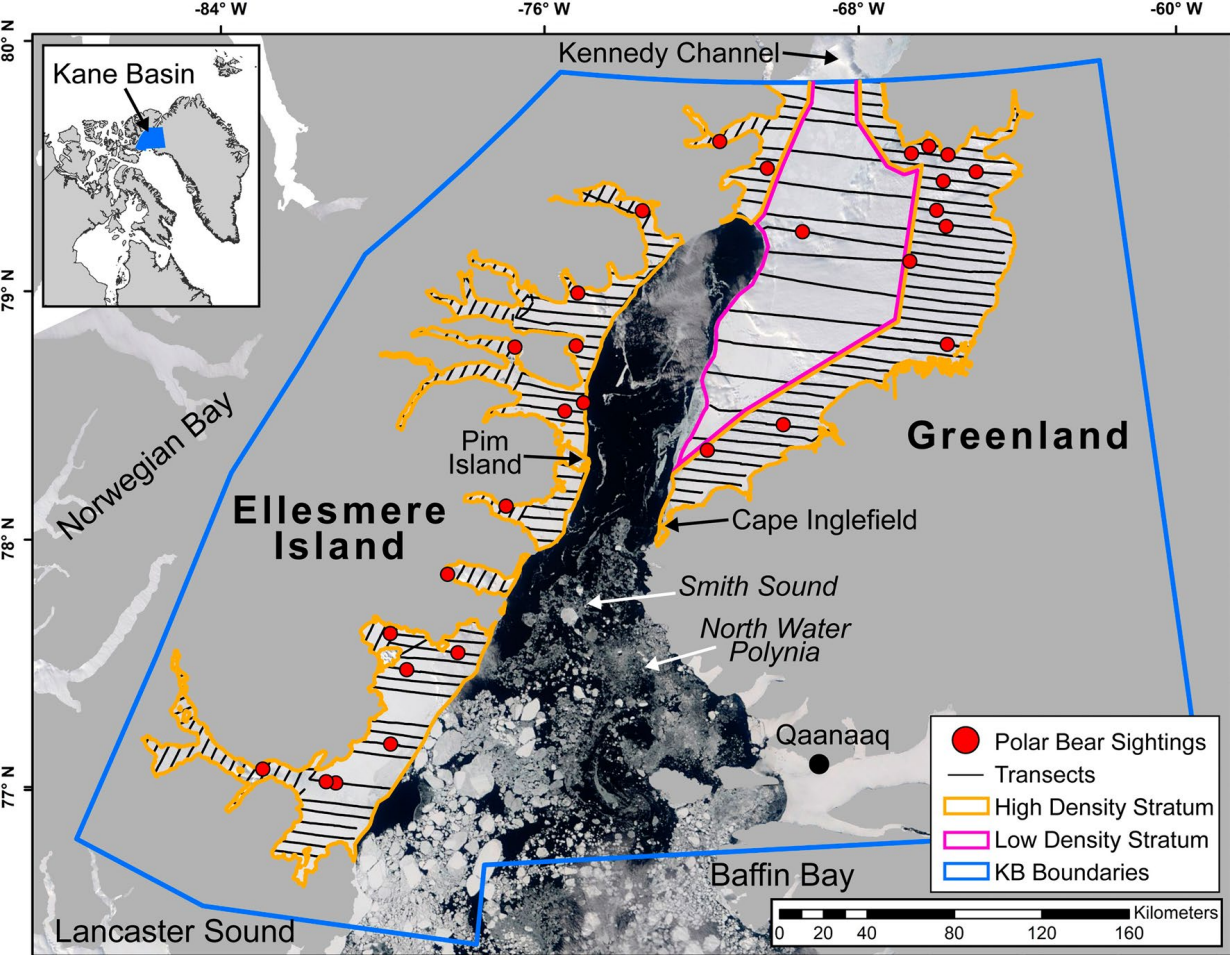
Transects surveyed and polar bear (*Ursus maritimus*) groups sighted during an aerial survey of the Kane Basin subpopulation during April–May, 2014. Transects and sightings are overlaid on a MODIS image (1 km resolution; available: http://modis.gsfc.nasa.gov/) collected on 5 May 2014. Sea ice in southeastern Kane Basin (i.e., to left of figure legend) was not sampled due to safety and logistical constraints presented by the North Water polynya and because we anticipated very low densities of polar bears (see text). Position of the Kane Basin subpopulation of polar bears in the Arctic is shown in upper left corner. Positions of surrounding subpopulations Norwegian Bay, Lancaster Sound and Baffin Bay are indicated

The KB subpopulation ranges over Kane Basin, Nares Strait, Smith Sound and adjacent fjords along eastern Ellesmere Island and Northwest Greenland, south of 80° 15′ N and north of 76° 45′ N on Ellesmere Island side and north of 77° N on Greenland side (PBSG 2018). The subpopulation is bounded to the north by the Arctic Basin subpopulation (via the Kennedy Channel), to the south by the Baffin Bay (BB) and Lancaster Sound (LS) subpopulations, and to the west by Norwegian Bay (NW; PBSG 2018; Fig. 1). Some limited interchange between KB and neighboring subpopulations has been demonstrated (Taylor et al. 2001; SWG 2016).

Sea ice remains in the northern range (i.e., Nares Strait–Kane Basin) throughout the year, largely due to the influx of polar pack ice from the Arctic Basin, and reaches a minimum in late summer. However, sea ice conditions have decreased markedly in the KB region in recent decades (Born et al. 2011; Stern and Laidre 2016; Laidre et al. 2020a). Laidre et al. (2020b) showed that the annual cycle of sea ice habitat in KB shifted from a year-round ice platform (~ 50% coverage in summer) in the 1990s to nearly complete melt-out in summer (< 5% coverage) in the 2010s. The North Water polynya (NOW), a large area of open water in northern Baffin Bay and southern Smith Sound, is a significant regional geographic feature that exhibits substantial intra- and inter-annual variability in spatial extent and is thought to form a barrier between KB and BB–LS subpopulations. Historically, the NOW had its northern limit at a distinct sea ice “bridge" from Cape Inglefield in NW Greenland across to Pim Island at Ellesmere Island in the northern part of Smith Sound (Barber et al. 2001). However, due to decrease in sea ice, this ice bridge has dissolved and the open water in NOW has stretched north into the Nares Strait–Kane Basin region during the last decades (Born et al. 2011; Heide-Jørgensen et al. 2013, 2016; Moore et al. 2021; Fig. 1). The extent of the NOW sensu stricto (i.e., area with open water or little and variable sea ice cover) during the 2014-survey is shown in Fig. 1.

### Field sampling

The aerial survey was a “platform of opportunity” that took advantage of a helicopter being used to complete the third year of a MR study (2012–2014), during which bears were remotely biopsied (2012–2014) and/or immobilized for handling (2012–2013) (SWG 2016). A single engine Bell 206 Long Ranger helicopter without pop-up floats and with an endurance of ca. 4 h and a maximal range of ca. 650 km was used in all years, which prevented us from surveying over broken ice and open water.

During the design stage, we stratified the subpopulation into high- and low density strata based on a priori observations of polar bears obtained during the 2012 and 2013 MR field operations. The high density stratum included landfast ice along the coastline and within fjords as well as nearshore pack ice within ~ 30 km of land (~ 18,870 km^2^). The low density stratum included offshore pack ice up to the outer edge of the open water in the NOW (~ 9110 km^2^; Fig. 1). We delineated the extent of available habitat by approximating the edge of the NOW with Moderate Resolution Imaging Spectroradiometer (MODIS; http://modis.gsfc.nasa.gov/) images (1 km resolution) using daily or near-daily imagery. The NOW’s polynya boundaries are variable, so we delineated the extent of the polynya adjacent to the section surveyed on a particular day using daily MODIS imagery, or from the closest date possible when same-day imagery was unclear due to atmospheric conditions. We also examined the delineated study area in relation to weekly regional sea ice charts produced by the Canadian Ice Service (https://www.ec.gc.ca/glaces-ice/). During sampling, we collected GPS waypoints at the edge of the polynya to verify delineation. We did not sample over the entire polynya (~ 27,214 km^2^; Fig. 1) due to safety considerations. We also did not sample the landfast sea ice in the fjords of the populated Qaanaaq area in NW Greenland (~ 3245 km^2^; Fig. 1), because few polar bears occur there due to intense hunting pressure by Greenlandic subsistence hunters (Taylor et al. 2001; Born et al. 2011).

We conducted a line transect aerial survey over the sea ice during 28 April–12 May 2014. During line transect sampling, we surveyed at an altitude of ~ 120 m and groundspeed of ~ 150 km/h. Aerial transects were systematically spaced at 6- and 18-km intervals in the high- and low density strata, respectively. We arranged transects in an east–west direction in open areas, but oriented them across the widths of fjords so that sighting distances would not be truncated by the sides of fjords (Fig. 1). When ferry flights between transects occurred over ice and were not associated with a habitat edge that might have affected bear densities (i.e., along the land or polynya edge, or the sides of fjords), we included these connecting flights as transects in our analyses.

We collected aerial survey data using a MR distance sampling (MRDS) protocol (Laake and Borchers 2004). Two front observers (including the pilot) and two rear observers comprised the first and second capture events, respectively. These teams worked independently until front and rear observers were each afforded a full opportunity to observe a bear. After announcing a sighting, we flew off-transect to record the location where bears were first observed using a GPS, and we later estimated perpendicular distance from transects in a GIS framework (Marques et al. 2006). During off-transect flights, we flew to within ca. 10 m of bears to obtain a tissue sample via biopsy darting for genetic analysis (SWG 2016) and to estimate sex, age class, and group size of the bear(s). We collected data on 4 covariates that potentially impact detection probability, including bear activity (moving vs. stationary), smooth versus rough ice (coded 0 vs. 1, respectively); visibility (good: 0 or compromised due to fog, glare or precipitation: 1), and cloud cover (in 25% quartiles).

### Statistical analyses

Data analysis used the MRDS package 2.2.0 (Laake et al. 2018) in Program R (R Development Core Team 2018). MRDS uses traditional distance sampling to estimate detection probability of clusters of organisms as a function of distance from the transect line (Buckland et al. 2001), but it also corrects for imperfect detection on the transect line using MR analysis (Laake and Borchers 2004). We defined clusters as discrete groups of bears with correlated detection probabilities (e.g. an adult female with 1 or more dependent offspring) and we treated individual clusters as sample observations. Because we measured exact distances to each cluster using GPS, we treated distance data as continuous (Buckland et al. 2001).

For MR analysis, we estimated detection probability ($\widehat{p}$) of each observed cluster (*i*) by observer position (*pos*) as:

1 $$logit\left( {\hat{p}_{{{\text{i}},{\text{pos}}}} } \right)\, = \,\hat{\beta }_{{0,{\text{pos}}}} \, + \,\hat{\beta }_{1} d_{{\text{i}}} \left[ { + \,\hat{\beta }_{2} X_{{\text{i}}} } \right]$$

We included position-specific intercepts (${\widehat{\beta }}_{0,\text{F}}$ or ${\widehat{\beta }}_{0,\text{R}}$) because previous analyses using this survey platform have documented higher detection probabilities for front- (F) versus rear- (R) seat observers (Stapleton et al. 2016; Conn and Alisauskas 2018), and we included perpendicular distance from the transect line (${\widehat{\beta }}_{1}{d}_\text{i}$) as a required covariate in all models (Laake et al. 2018). Due to limited sample size (28 clusters), we considered a maximum of one additional covariate (Giudice et al. 2012). We allowed the effect of distance to vary by observer position (i.e., by fitting a unique slope ${\widehat{\beta }}_{1,\text{pos}}$ for front versus rear observers). We also considered squared distance, cluster size, bear activity, ice conditions, visibility, and cloud cover as potential covariates affecting both front and rear observer positions equally. Finally, we created a dummy covariate (*X*_i,pos_) by coding detections that were within 75 m of the transect line for rear observers as 1 (this represents a potential blind spot for rear observers using this survey platform; Stapleton et al. 2016), with all remaining covariate values coded as 0. The product of ${\beta }_{2}{X}_{i,\text{pos}}$ is therefore the estimated reduction in detection probability on the logit scale for rear seat observers when bears were in the blind spot directly below the helicopter and 0 in all other situations. Our a priori model set therefore included 9 potential models, which we ranked using second-order Akaike’s information criterion AIC_c_ (Burnham and Anderson 2002).

The probability of at least one observer detecting a cluster located at distance 0 from the transect line (${\widehat{p}}_{0}^{*}$) is one minus the product that both observers will miss the cluster, or:

2 $$\hat{p}_{0}^{*} \, = \,1\, - \,\left( {1\, - \,\hat{p}_{{{\text{F}},0}} } \right)*(1\, - \,\hat{p}_{{{\text{R}},0}} )$$

In the event of model-uncertainty, we model-averaged predicted values of ${\widehat{p}}_{0}^{*}$ using models with ∆AIC_c_ < 4, unless these models included uninformative parameters (Burnham and Anderson 2002; Arnold 2010).

For modeling distance sampling data, we considered all standard key functions including a uniform key function with cosine or standard polynomial adjustment terms, a hazard-rate key function, and a half-normal key function (Miller et al. 2016). We considered additional and higher-order cosine, polynomial, or Hermite polynomial adjustment terms for each key function if they were supported by lower AIC (Miller et al. 2016), otherwise we treated additional adjustment terms as uninformative parameters. We also considered cluster size as a potential covariate for each key function. In the event of model selection uncertainty (i.e., multiple competitive distance functions with ∆AIC_c_ < 4), we model-averaged estimates of mean detection probability within the transect boundaries (${\widehat{p}}_\text{d}$). Average detection probability (${\widehat{p}}_\text{a}$) was the product of ${\widehat{p}}_{0}^{*}$ and ${\widehat{p}}_\text{d}$, with variance estimated using the delta method:

3 $$\widehat{var}\left({\widehat{p}}_{a}\right)\,=\,{\left({\widehat{p}}_{0}^{*}\right)}^{2}\left(\widehat{var}\left[{\widehat{p}}_{d}\right]\right)\,+\,{\left({\widehat{p}}_{d}\right)}^{2}\left(\widehat{var}\left[{\widehat{p}}_{0}^{*}\right]\right)$$

Abundance of polar bear clusters in the surveyed area (${\widehat{C}}_\text{S}$) was estimated using a Horvitz-Thompson-like estimator (Miller et al. 2016):

4 $${\widehat{C}}_\text{S}\,=\sum_{s=1}^{2}\frac{{c}_\text{k}}{{\widehat{p}}_\text{a}}$$

where *k* indicates stratum 1 or 2, ${c}_\text{k}$ is the number of clusters detected in stratum *k*, and ${\widehat{p}}_\text{a}$ is average detection probability. Variance due to detection probability (${\widehat{var}}_\text{detection}{\widehat{C}}_\text{S}$) is given by:

5 $${\widehat{var}}_\text{detection}\left({\widehat{C}}_\text{S}\right)=\sum_{k=1}^{2}(-{c}_\text{k}{*{\widehat{p}}_\text{a}}^{-2}{)}^{2}*\widehat{var}({\widehat{p}}_\text{a})$$

which can be scaled up to the entire study area (${\widehat{C}}_\text{Total}$) based on survey coverage (A: stratum area/a: survey area) within each stratum (e.g. Powell 2007):

6 $${\widehat{var}}_\text{detection}\left({\widehat{C}}_\text{Total}\right)=\sum_{k=1}^{2}{\left(\frac{{A}_\text{k}}{{a}_\text{k}}\right)}^{2}(-{c}_\text{k}{*{\widehat{p}}_{a}}^{-2}{)}^{2}*\widehat{var}({\widehat{p}}_\text{a})$$

We considered each transect the sampling unit for estimating encounter rate variance, ${\widehat{var}}_\text{encounter}\left({\widehat{C}}_\text{Total}\right)$, and we used variance estimation method S2 within program MRDS, which recognizes systematic placement of transects by treating adjacent transects as paired samples and also accounts for variation in length among transects (Fewster et al. 2009). Total variance of clusters was the sum of detection rate variance and encounter rate variance:

7 $$\widehat{var}\left({\widehat{C}}_\text{Total}\right)={\widehat{var}}_\text{detection}\left({\widehat{C}}_\text{Total}\right)+{\widehat{var}}_\text{encounter}\left({\widehat{C}}_\text{Total}\right)$$

For estimates of total abundance ($\widehat{N}$), we multiplied estimated number of clusters (${\widehat{C}}_\text{Total}$) by mean group size per cluster ($\widehat{g}$), with variance estimated as:

8 $$\widehat{var}\left({\widehat{N}}_\text{Total}\right)={\left({\widehat{C}}_\text{Total}\right)}^{2}\widehat{var}\left(\widehat{g}\right)+{\left(\widehat{g}\right)}^{2}\widehat{var}\left({\widehat{C}}_\text{Total}\right)$$

Although distance and MRDS programs both perform delta method variance estimates internally, we estimated variance components separately using Eqs. 3–8 to utilize model-averaged estimates of ${\widehat{p}}_{0}^{*}$ and ${\widehat{p}}_\text{d}$, and to allow flexibility in choosing stratum- or survey-wide estimates of parameters. Given only one cluster was detected in the low density stratum, we constrained ${p}_{0}^{*}$ distance functions, and mean cluster size to be identical across strata, allowing only encounter probability (and its variance) to differ between strata. For model-averaged estimates, we also included model selection uncertainty as a component of variance (Burnham and Anderson 2002: Eq. 4.9). We extrapolated our model-averaged density estimate from the low density stratum to the sea ice near Qaanaaq (3245 km^2^) in southeastern KB and for the unsampled sea ice-covered central parts of the North Water polynya (27,214 km^2^) where we know from experience some bears might occur and estimated log normal 95% CIs.

## Results

**Fig. 2:**
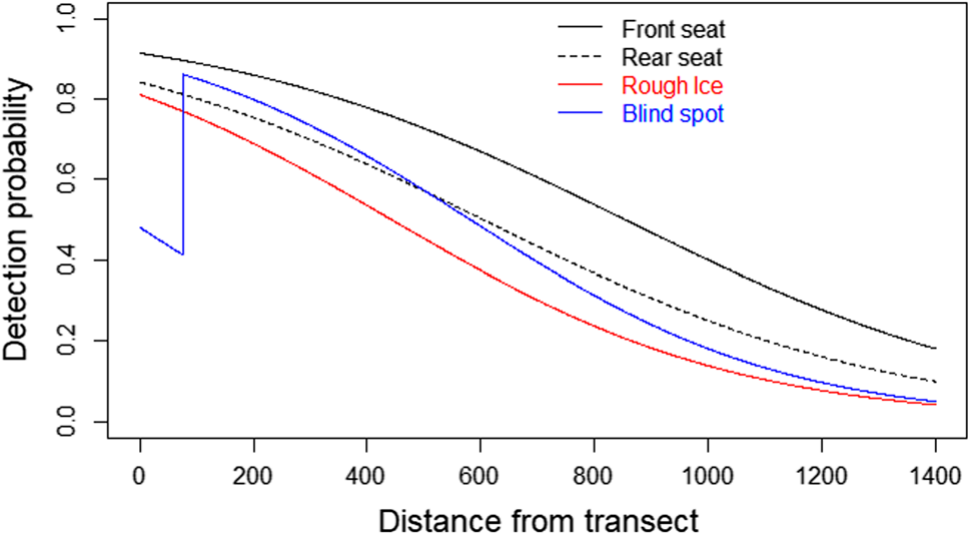
Estimated detection probabilities of polar bear (*Ursus maritimus*) clusters to front- and rear seat observers as a function of distance from transect line, as estimated from the mark-recapture submodel of program MRDS. The effect of rough ice (red line) is plotted for the front-seat observer, whereas the effect of reduced visibility out to 75 m is plotted for the rear seat observer. Note that rear seat observers detected 1 of 3 available bears at 0–75 m, so detection probability was not 0 in this range

We flew a total of 70 h and surveyed 4160 km of transects, including 3389 km along 222 a priori transects in the high density stratum, 681 km along 14 transects in the low density stratum, and 90 km along 9 ferry transects in the high density stratum. Bears were observed consistently out to 1400 m on each side of the helicopter (Fig. 2), and one bear 3588 m off transect, so we used 1400 m as our truncation distance to improve model performance (Buckland et al. 2001). After truncation, we retained 28 clusters of polar bears for analysis, including 26 clusters along a priori transects in the high density stratum, 1 on ferry transects, and 1 on low density transects (Fig. 1).

Neither mark-resighting nor distance-based estimates of detection probability were influenced by cluster size (AIC increased by 0.2 to 2.0 units whenever cluster size was included as a covariate), so we used mean cluster size for abundance estimation. Cluster size averaged 1.75 bears (SE 0.15), and included 13 lone bears (2 adult females, 5 adult males, 3 subadult males, and 3 subadults of unknown sex), 9 groups of two (5 females with a single cub-of-the year, 1 female with a yearling cub, 1 female with a 2-year-old, and 2 pairs of adult bears), and 6 groups of three (all cases involved adult females with twin cubs-of-the-year). Cub-of-the-year litter size averaged 1.55 (6 twins, 5 singletons).

Front-seat observers detected 22 of 28 observed clusters, whereas rear seat observers detected 16 clusters (Table 1), with modeled detection probability declining monotonically for both observers (Fig. 2). Models that included parameters for ice structure (smooth vs. rough) and reduced visibility for rear seat observers out to 75 m explained additional variation in detection probability and were supported by lower AIC_c_ (Table 2; Fig. 2). All three AIC-supported MR models fit the data (χ^2^ < 13.15, 10 or 11 df, *P* > 0.22), with pooled detection probability on the transect line (${\widehat{p}}_{0}^{*}$) of 0.932 (SE 0.077) to 0.975 (SE 0.029), with a model-averaged estimate of 0.953 (SE 0.056) for clusters of bears located directly on the transect line (Table 2).

**Table 1:** Frequency of sightings (Seen) and sighting failures (Missed) of polar bear (*Ursus maritimus*) clusters by front (F) and rear (R) seat observers in different distance bins (m) during on-ice aerial mark-recapture distance sampling surveys conducted in Kane Basin, April–May, 2014

Distance bin	Seen F	Missed F	Seen R	Missed R	Seen both	Total
0–200	8	0	4	4	4	8
200–400	6	0	3	3	3	6
400–600	3	2	4	1	2	5
600–800	1	1	2	0	1	2
800–1000	1	2	2	1	0	3
1000–1200	1	1	1	1	0	2
1200–1400	2	0	0	2	0	2
Combined	22	6	16	12	10	28

**Table 2:** Results of model selection for the mark-recapture component of a mark-recapture distance sampling (MRDS) survey of polar bears (*Ursus maritimus*) in Kane Basin, April–May, 2014

Additions	∆AIC_c_	*w*_i_	${\widehat{p}}_{\text{F},0}$	SE (${\widehat{p}}_{\text{F},0}$)	${\widehat{p}}_{\text{R},0}$	SE (${\widehat{p}}_{\text{F},0}$)	${\widehat{p}}_{0}^{*}$	SE (${\widehat{p}}_{0}^{*}$)
Ice structure	0.00	0.41	0.826	0.116	0.720	0.142	0.932	0.077
Blind spot	0.35	0.34	0.891	0.080	0.774	0.113	0.975	0.029
None	0.98	0.25	0.842	0.097	0.727	0.133	0.957	0.044
Model avg			0.852	0.103	0.740	0.132	0.953	0.056

All models included intercept, observer, and distance effects (3 *df*) with up to one additional covariate; models with additional uninformative parameters not shown. ∆AIC_c_ is difference in Akaike’s information criterion between listed model and top-ranked model, *w*_i_ is Akaike weight, ${\widehat{p}}_{\text{obs},0}$ and SE(${\widehat{p}}_{\text{obs},0}$) is the probability and associated standard error of observing a cluster of polar bears for front- (F) and rear seat (R) observers at a sighting distance of zero meters from the transect line, and ${\widehat{p}}_{0}^{*}$ is the probability that a cluster located on the transect line will be detected by at least one observer

AIC_c_ of the top-ranked model was 59.35

**Fig. 3:**
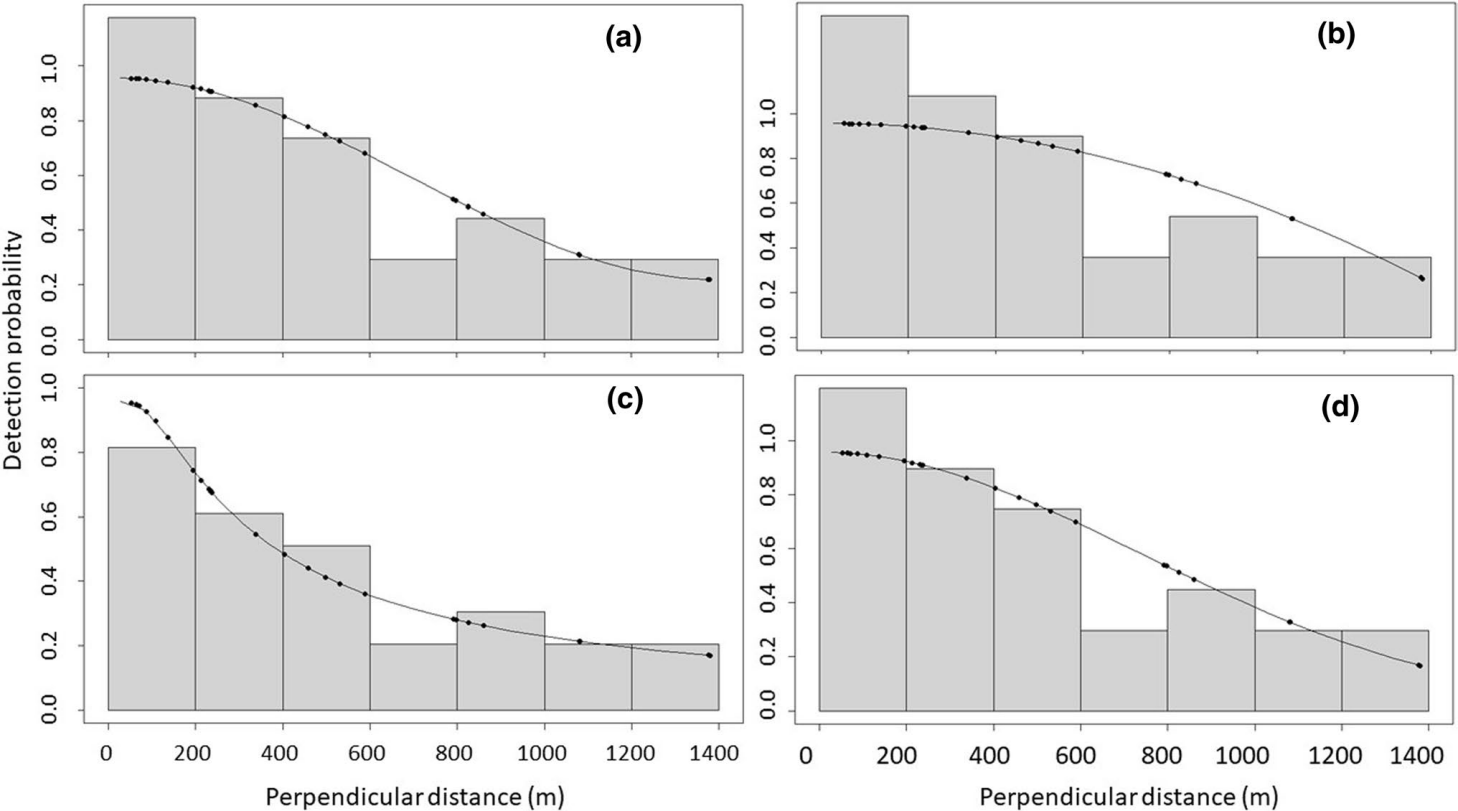
Histograms summarizing sighting distances and estimated detection functions **a** Uniform cosine, **b** Uniform polynomial, **c** Hazard rate, **d** Half normal from an aerial survey of the Kane Basin polar bear (*Ursus maritimus*) subpopulation, April–May, 2014. See Table 3 for model statistics

There was considerable uncertainty in estimation of detection functions for bears off the transect line (Fig. 3), with 4 different key functions fitting the data and showing reasonable support (∆AIC ≤ 3; Table 3). The hazard-rate model estimated the lowest average detection probability ($\widehat{p}$ = 0.426, SE = 0.198), whereas half-normal and uniform functions with adjustment terms estimated greater detection probabilities of 0.585–0.716 with less uncertainty (Table 3). Model-averaged detection probability from distance sampling was 0.571 (SE 0.151) and when combined with MR sampling, detection probability averaged 0.547 (SE 0.147). Because the coefficient of variation for the hazard detection function was nearly 3 times larger than for the other three functions, we also model-averaged results while excluding the hazard function, and estimated overall detection probability of 0.610 (SE 0.091).

**Table 3:** Detection functions fit to polar bear (*Ursus maritimus*) distance sampling data from Kane Basin, April–May, 2014

Key function	CvM	*P*	df	∆AIC^a^	w_i_	${\widehat{p}}_\text{d}$	SE (${\widehat{p}}_\text{d}$)	$\widehat{p}$	SE ($\widehat{p}$)^b^
Uniform-cos	0.13	0.45	4	0.00	0.356	0.614	0.077	0.585	0.081
Hazard-rate	0.04	0.94	5	0.27	0.311	0.426	0.198	0.406	0.190
Half-normal	0.14	0.42	4	0.84	0.234	0.623	0.088	0.594	0.091
Uniform-poly	0.31	0.12	4	2.58	0.098	0.751	0.070	0.716	0.079
Model avg. 4						0.571	0.151	0.544	0.147
Model avg. 3						0.637	0.091	0.610	0.091

Columns include key functions, Cramér-von Mises (CvM) test statistics and associated P-values (where low P-values indicate lack of fit), number of model parameters (df), Akaike’s information criterion (∆AIC) and AIC weight (w_*i*_), mean detection probability (${\widehat{p}}_\text{d}$) and SE(${\widehat{p}}_\text{d}$) from distance sampling analysis, and combined detection probability ($\widehat{p}$) and SE ($\widehat{p}$) from joint MRDS analysis. Model-averaged estimates are based on results from all 4 models (Model avg. 4) and results with the Hazard-rate model excluded (Model avg. 3)

^a^AIC of top-ranked model = 400.89

^b^SE of combined detection probability determined via delta method (Eq. 3) using model-averaged estimate of ${\widehat{p}}_{0}^{*}$ from Table 2

Estimates of total abundance of bears varied greatly among models, ranging from 139 to 170 for models using uniform or half-normal key functions, versus 245 for the hazard-rate model (Table 4). Our model-averaged estimate of total abundance was 190 bears (95% lognormal CI: 87–411; CV 39%) based on all 4 models, but 165 bears (95% lognormal CI: 101–269; CV 24%) with the hazard-rate model excluded (Table 4). Most of the variation in abundance was attributable to uncertainty in estimating detection functions (hazard-rate function and model-averaged results) or encounter rates (uniform and half-normal detection functions), with minimal uncertainty due to estimating group size or detection on the transect line (Table 5). Extrapolating our model-averaged density estimate for all four models from the low density stratum [1.7 (SE 1.9) bears per 1000 km^2^] to unsurveyed sea ice near Qaanaaq in southeastern KB yielded ~ 6 (95% lognormal CI 1–36) bears and for the unsampled central parts of the North Water polynya yielded ~ 46 (CI 7–293) bears.

**Table 4:** Estimated densities and abundances of the Kane Basin polar bear (*Ursus maritimus*) subpopulation, April–May, 2014, based on four mark-recapture distance sampling models

Model	*w*_i_	Density (bears/1000 km^2^)						Abundance		
		High	SE	Low	SE	Total	SE	$\widehat{N}$	SE	CV
Uniform-cos	0.356	8.2	1.9	1.6	1.6	6.1	1.4	170	40	0.23
Hazard-rate	0.311	11.9	6.0	2.3	2.5	8.8	4.4	245	124	0.50
Half-normal	0.234	8.1	1.9	1.5	1.6	6.0	1.4	167	40	0.24
Uniform-poly	0.098	6.7	1.4	1.3	1.3	5.0	1.1	139	30	0.22
Model avg. 4		9.2	3.6	1.7	1.9	6.8	2.7	190	74	0.39
Model avg. 3		8.0	1.9	1.5	1.6	5.9	1.4	165	40	0.24

Densities are expressed per 1000 km^2^ of surveyed sea ice (357 transect km) and are therefore unique to sea ice conditions that occurred during our survey. High and low refer to stratum-specific estimates of density (see Fig. 1). Model-averaged estimates (last two rows) are based on AIC model weights (*w*_*i*_), with Model avg. 4 including results from all 4 models and Model avg. 3 excluding results from the less certain hazard-rate model

**Table 5:** Coefficients of variation (CV) in individually estimated components of abundance for Kane Basin polar bears (*Ursus maritimus*) based on on-ice mark-recapture distance sampling surveys conducted during April–May, 2014

Model	${\widehat{p}}_{0}^{*}$	${\widehat{p}}_\text{d}$	${\widehat{p}}_\text{a}$	${\widehat{p}}_\text{Enc}$	${\widehat{C}}_\text{Total}$	$\widehat{g}$	${\widehat{N}}_\text{Total}$
Uniform cos1	0.06	0.13	0.13	**0.16**	0.22	0.09	0.24
Hazard-rate	0.06	**0.46**	0.47	0.16	0.50	0.09	0.51
Half-normal	0.06	0.14	0.15	**0.16**	0.23	0.09	0.24
Uniform poly2	0.06	0.09	0.10	**0.16**	0.20	0.09	0.22
Model avg. 4	0.06	**0.26**	0.27	0.16	0.38	0.09	0.40
Model avg. 3	0.06	0.14	0.15	**0.16**	0.23	0.09	0.24

Variance components include mark-recapture based probability of detection on the transect line (${\widehat{p}}_{0}^{*}$), detection function probability based on distance sampling (${\widehat{p}}_\text{d}$), combined MRDS detection probability (${\widehat{p}}_\text{a}$), encounter rate variation (${\widehat{p}}_\text{Enc}$), cluster abundance (${\widehat{C}}_\text{Total}$), mean group size per cluster ($\widehat{g}$), and total population size (${\widehat{N}}_\text{Total}$). For each model, the largest individual component of variation is highlighted in bold

## Discussion

Aerial surveys for monitoring trends in distribution and abundance are routinely used for Arctic marine mammals with large geographic distributions including white whales (*Delphinapterus leucas)*, narwhals (*Monodon monoceros),* walrus (*Odobenus rosmarus),* bearded seals, ringed seals, and polar bears (e.g. Innes et al. 2002; Aars et al. 2009, 2017; Estes and Gilbert 2010; Heide-Jørgensen et al. 2013, 2016; Vacquié-Garcia et al. 2017, 2020; Bröker et al. 2019; Conn et al. 2021).

A minimum of 60–80 observations are recommended when using the distance sampling method to ensure reliable detection function estimation (Buckland et al. 2001). Our aerial survey estimate of polar bears was based on a smaller number of observations (*n* = 28) due to low densities of bears and logistical constraints on sampling effort, resulting in greater uncertainty in estimation of the detection functions, particularly with the hazard-rate model which had a much greater coefficient of variation. We support the use of the model-averaged estimate of total abundance based on all 4 models (190 bears, 95% lognormal CI: 87–411, Table 4) because it incorporates this model selection uncertainty, but note that estimated density would be markedly lower and more precise if this model was omitted. Previous applications of aerial distance sampling for polar bears have found support for both half-normal and hazard-rate detection functions (Stapleton et al. 2014, 2016; Conn et al. 2021).

Our estimate of abundance in KB is likely negatively biased because we did not sample major portions of the KB subpopulation’s potential distribution area because of logistical and safety considerations (Fig. 1); however, expected bear densities in unsampled regions were extremely low as indicated by earlier spring aerial surveys of the NOW (Heide-Jørgensen et al. 2013, 2016). For example, during multi-species aerial surveys to determine distribution and abundance of marine mammals in the NOW area in 2009 and 2010, only seven polar bears were observed (2009:2, 2010:5); five of which were detected in areas that were also covered during our survey (i.e. western sector of the NOW and in the Nares Strait/KB region) (Heide-Jørgensen et al. 2013, 2016). During 10 and 14 April 2014, a systematic aerial survey to determine abundance of marine mammals was conducted over the eastern parts of the NOW between 76° and 78° N, and 72° 45′ and 76° W (approximately 16,000 km^2^), but no polar bears were reported (Heide-Jørgensen et al. 2016). It is well known that multi-species aerial surveys are not ideal for detecting polar bears because of the large difference in visual cues between bears on sea ice and other marine mammals occurring in water among ice floes. However, these surveys, in addition to only one observation over the low density area in our study highlight the general scarcity of bears in sectors of the NOW and suggest that the number of bears missed by not surveying areas with sub-optimal polar bear habitat was likely low.

The unsampled sea ice regions covered large areas (sea ice near Qaanaaq and the North Water polynya: 30,459 km^2^), such that even very low densities could have had a significant contribution the overall estimate of abundance. However, given the very high uncertainty in the estimates of number of bears in these regions, we suggest that the negative bias arising from incomplete sampling of the ice-covered areas of the KB polar bear subpopulation cannot be determined with any certainty. As the survey was conducted in late April-early May after adult female polar bears and their dependent young had emerged from maternal dens (Escajeda et al. 2018), we believe that few bears remained on land during the survey period so that our estimate is a reasonable estimate for the KB subpopulation.

The ability of distance sampling to generate unbiased abundance estimates is dependent on four critical assumptions (Buckland et al. 2001). We surveyed with systematically spaced transects oriented perpendicular to the coastal density gradient to satisfy the first assumption that organisms would be randomly distributed with respect to distance from the transect line (Stapleton et al. 2014). A second critical assumption of distance sampling is that the probability to observe organisms directly on the transect line equals 1 (Buckland et al. 2001). We evaluated this assumption with double-observer models and estimated ${\widehat{p}}_{0}^{*}$ = 95.3%, which suggests that virtually all animals on the transect line were observed (moreover, MRDS automatically corrects for any violation of this assumption). A third assumption is that all organisms are observed at their initial location (i.e. before responding to approaching aircraft). Only 4 of 29 observations involved bears that were running when first observed; the remaining 25 groups were standing, sitting, or walking, suggesting that most observations were unaffected by potential movement off transect. Although we cannot rule out the possibility that bears first observed running were responding to aircraft noise, our flight speed was rapid (approx. 150 km/h) and would have minimized the opportunity for bears to move very far before detection. Finally, accurate measurement of distances to sightings from the transect path is critical (Buckland et al., 2001). We used methods involving GPS and GIS technology adapted from Marques et al. (2006) that have been widely used in polar bear aerial surveys (e.g. Aars et al. 2009, 2017; Stapleton et al., 2014, 2016) and are therefore confident that our measures of perpendicular distance between the aircraft flight path and polar bears were accurate.

Additional minor assumptions of distance sampling include accurate estimates of group size, avoidance of double counting, and independence of observations (Buckland et al. 2001). Because we conducted our survey over open ice, we believe that all group counts were accurate. Likewise, double counting is unlikely given rapid survey speed and wide transect spacing (6 or 18 km), relative to the speed polar bears travel over short time periods (e.g. average travel speed of polar bears 1.5 to 3.5 km per hour; Durner et al. 2017: Table 2). Because our aerial survey occurred in year 3 of a MR study, one reviewer expressed concern that previously marked bears may have tried to avoid approaching aircraft. However, we do not believe that trap shyness is an important factor in aerial surveys of polar bears given most bears were stationary or walking when first observed. Fieberg et al. (2015) conducted a meta-analysis of potential behavioral responses of previously marked ungulates during aerial surveys and likewise concluded there were no behavioral effects. Furthermore, physical sampling that occurred during the 2014 aerial survey in KB consisted of biopsy darting, not physical captures. The average time between sighting a bear and collecting a biopsy sample is 2–5 min (according to our experience), which means that other bears in the area will experience a short disturbance and have little time to leave the area.

A general concern with any aerial survey estimate of widely distributed Arctic marine mammals occurring in low densities, is that they generally have a relatively low precision (high CV). McDonald et al. (1999) regarded an aerial survey of polar bears to be robust if the CV was 25% or less. Because precision improves as 1/√n, reducing our observed CV of 39% to 25% or lower would require ($0.39/0.25{)}^{2}\approx 2.4$-fold additional sampling effort. Increasing the survey effort in KB by this factor from 4120 to ca. 9984 linear km of effort would require ca. 39 h of additional search effort at a survey speed of 150 km/h, which seems to be realistically achievable. However, transect spacing less than 3 km apart to achieve higher precision of the estimate could become an issue in a small high density ice area due to disturbance and movement of bears between transect lines during a survey. Future polar bear aerial surveys should be designed as single-species surveys and should consider using fixed-winged aircraft perhaps in combination with a helicopter if feasible (SWG 2011).
